# Identifying the ‘Active Ingredients' of an Effective Psychological Intervention to Reduce Fear of Cancer Recurrence: A Process Evaluation

**DOI:** 10.3389/fpsyg.2021.661190

**Published:** 2021-06-07

**Authors:** Janice M. Kan, Mbathio Dieng, Phyllis N. Butow, Shab Mireskandari, Stephanie Tesson, Scott W. Menzies, Daniel S. J. Costa, Rachael L. Morton, Graham J. Mann, Anne E. Cust, Nadine A. Kasparian

**Affiliations:** ^1^Discipline of Paediatrics, School of Women's and Children's Health, UNSW Medicine, The University of New South Wales, Sydney, NSW, Australia; ^2^NHMRC Clinical Trials Centre, The University of Sydney, Sydney, NSW, Australia; ^3^Centre for Medical Psychology and Evidence-Based Decision-Making, School of Psychology, The University of Sydney, Sydney, NSW, Australia; ^4^Psycho-Oncology Co-operative Research Group, School of Psychology, The University of Sydney, Sydney, NSW, Australia; ^5^Discipline of Dermatology, Sydney Medical School, The University of Sydney, Sydney, NSW, Australia; ^6^The Sydney Melanoma Diagnostic Centre, Royal Prince Alfred Hospital, Sydney, NSW, Australia; ^7^Pain Management Research Institute, The University of Sydney at Royal North Shore Hospital, Sydney, NSW, Australia; ^8^School of Psychology, University of Sydney, Sydney, NSW, Australia; ^9^Melanoma Institute Australia, The University of Sydney, Sydney, NSW, Australia; ^10^John Curtin School of Medical Research, College of Health and Medicine, The Australian National University, Canberra, ACT, Australia; ^11^Cancer Epidemiology and Prevention Research, Sydney School of Public Health, The University of Sydney, Sydney, NSW, Australia; ^12^Cincinnati Children's Center for Heart Disease and Mental Health, Heart Institute and the Division of Behavioral Medicine & Clinical Psychology, Cincinnati Children's Hospital, Cincinnati, OH, United States; ^13^Department of Pediatrics, University of Cincinnati College of Medicine, Cincinnati, OH, United States

**Keywords:** fear cancer recurrence, intervention, melanoma, survivorship, psychological stress, process evaluation

## Abstract

**Purpose:** Psychological interventions targeting fear of cancer recurrence (FCR) are effective in reducing fear and distress. Process evaluations are an important, yet scarce adjunct to published intervention trials, despite their utility in guiding the interpretation of study outcomes and optimizing intervention design for broader implementation. Accordingly, this paper reports the findings of a process evaluation conducted alongside a randomized controlled trial of a psychological intervention for melanoma patients.

**Methods:** Men and women with a history of Stage 0–II melanoma at high-risk of developing new primary disease were recruited *via* High Risk Melanoma Clinics across Sydney, Australia and randomly allocated to receive the psychological intervention (*n* = 80) or usual care (*n* = 84). Intervention participants received a tailored psycho-educational resource and three individual psychotherapeutic sessions delivered *via* telehealth. Qualitative and quantitative data on intervention context, processes, and delivery (reach, dose, and fidelity), and mechanisms of impact (participant responses, moderators of outcome) were collected from a range of sources, including participant surveys, psychotherapeutic session audio-recordings, and clinical records.

**Results:** Almost all participants reported using the psycho-educational resource (97%), received all intended psychotherapy sessions (96%), and reported high satisfaction with both intervention components. Over 80% of participants would recommend the intervention to others, and a small proportion (4%) found discussion of melanoma-related experiences confronting. Perceived benefits included enhanced doctor-patient communication, talking more openly with family members about melanoma, and improved coping. Of potential moderators, only higher FCR severity at baseline (pre-intervention) was associated with greater reductions in FCR severity (primary outcome) at 6-month follow-up (primary endpoint).

**Conclusions:** Findings support the acceptability and feasibility of a psychological intervention to reduce FCR amongst individuals at high risk of developing another melanoma. Implementation into routine melanoma care is an imperative next step, with FCR screening recommended to identify those most likely to derive the greatest psychological benefit.

## Introduction

Fear of cancer recurrence (FCR) is one of the most common psychological difficulties experienced by people with cancer, and over 70% of people with a history of melanoma report clinically concerning levels (Costa et al., 2016). FCR is described as persistent worry and uncertainty about the possibility of developing new or recurrent disease, and is associated with poorer psychological well-being (Koch et al., 2013; Mutsaers et al., 2016), self-care, and health-related quality of life (Crist and Grunfeld, 2013; Simard et al., 2013; Lebel et al., 2020), as well as increased health service use and costs (Lebel et al., 2013; Simard et al., 2013). While a substantial proportion of individuals with melanoma report FCR and unmet emotional needs (Kasparian, 2013; Beesley et al., 2015; Stamataki et al., 2015; Costa et al., 2016; Fu et al., 2020), few receive professional psychological support (McLoone et al., 2012).

To address this gap in clinical care, our group developed a novel intervention comprising two components designed for people at high risk of developing new primary melanoma: (a) a tailored psycho-educational resource in booklet format, and (b) three individual psychotherapeutic sessions delivered *via* telehealth (Dieng et al., 2015, 2017; Kasparian et al., 2016). We tested the efficacy of this intervention in a longitudinal randomized controlled trial (RCT). Compared to those who received usual care, participants who received the intervention reported significantly lower FCR severity and psychological distress, fewer triggers to FCR, and improved melanoma-related knowledge at 6 months post-intervention (Dieng et al., 2016b). Moreover, reductions in FCR severity were sustained at 12-month follow-up (Dieng et al., 2020), and the study was found to be cost-effective and reasonable value for money in reducing FCR (Dieng et al., 2019).

While these results are positive, the multiple components of our intervention raises questions about the “active ingredients” contributing to successful outcomes. During study design, planning, and implementation, several factors were hypothesized to contribute to the potential effects of the intervention, such as greater time spent engaging with the psychologist (i.e., longer telehealth sessions), reading the psycho-educational resource more thoroughly, and increased knowledge about melanoma. To examine these hypotheses, we carried out a process evaluation alongside our longitudinal RCT.

**Figure 1:**
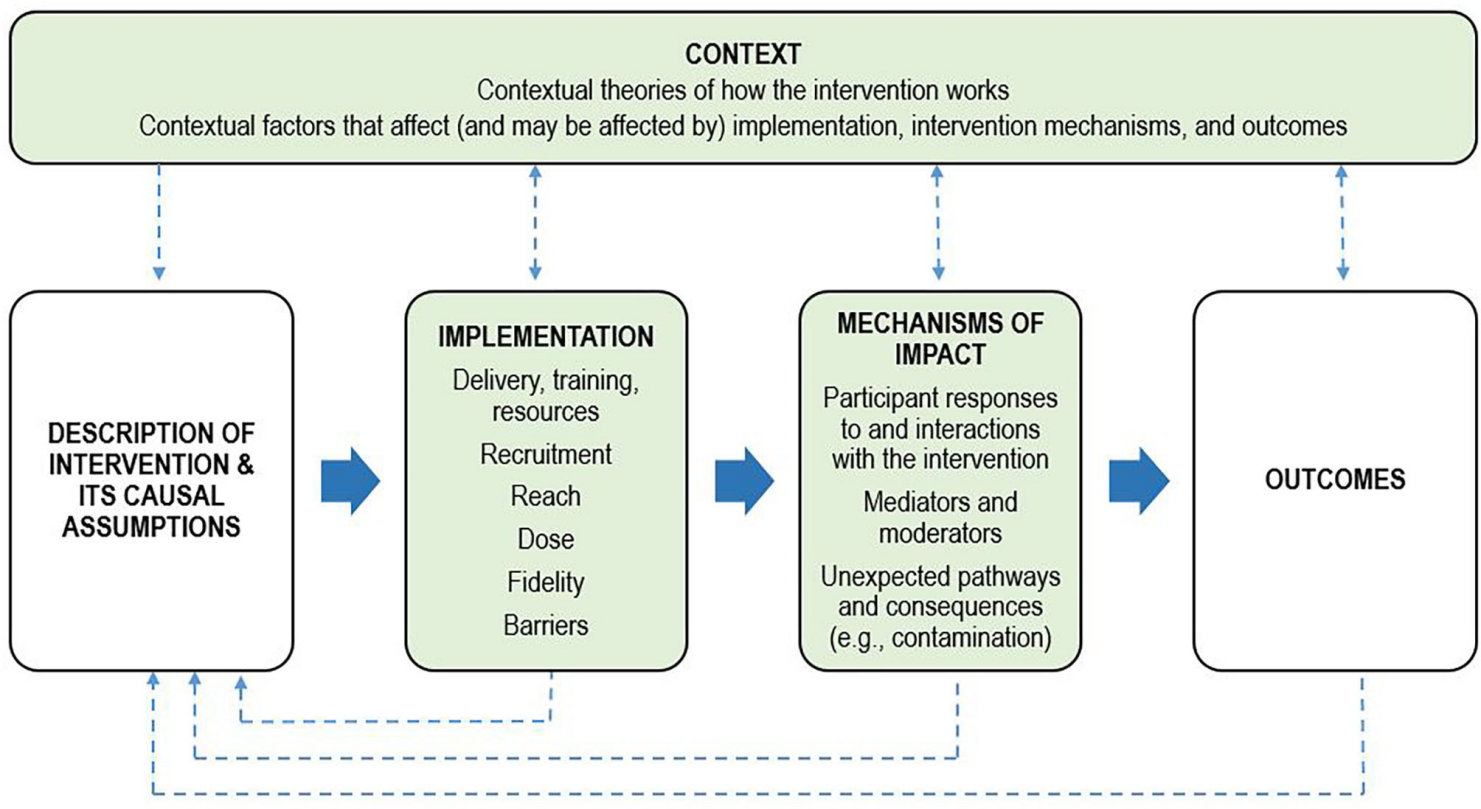
Process evaluation framework. Adapted from the UK Medical Research Council framework for conducting and reporting process evaluation studies (Moore et al., 2015). Key components of the process evaluation are in green boxes. Investigation of these components is shaped by clear descriptions of the intervention and its causal assumptions. Implementation refers to how the intervention was delivered, and mechanisms of impact refers to how the intervention produced change. The dotted lines indicate the relations between context, the intervention, implementation, mechanisms, and outcomes.

Process evaluations are an important, yet scarce adjunct to published intervention trials and aim to identify potential barriers and facilitators to translation. Briefly, process evaluations examine the quantity and quality of what was implemented during an intervention trial, how and by whom, and provide data to support or augment the interpretation of outcomes (Moore et al., 2014). Across psycho-oncology, there is a dearth of published process evaluations of psychological interventions, limiting our ability to effectively translate research findings into clinical practice and address unmet mental health needs. Key functions and components of process evaluations have been described (Moore et al., 2015), with the UK Medical Research Council framework providing a comprehensive guide (Figure 1). Process data can assist with interpreting intervention outcomes as well as inform the refinement of existing interventions and guide implementation by providing information on reach (who received the intervention), dose (what was delivered to, and received by, participants), fidelity (the extent to which the intervention was implemented in line with the protocol), and how participants perceived the intervention (satisfaction and helpfulness). Process data can also highlight barriers and contextual factors related to the environment in which the intervention was delivered that could influence outcomes. Therefore, the aims of this process evaluation were to:

a. Examine implementation (reach, fidelity, dose, and context) of an effective psychological intervention targeting fear of cancer recurrence amongst people at high-risk of developing new primary melanoma, as well as potential mechanisms of impact;
b. Assess participants' perceptions of the intervention, including acceptability and satisfaction; and
c. Provide data to assist in interpreting trial outcomes, as well as how best to implement the intervention in the future to maximize benefits and minimize risks in settings where ongoing dermatologic care is provided for people with melanoma.

## Materials and Methods

### Design and Measurement Approach

This process evaluation was conducted alongside a longitudinal randomized controlled trial. Intervention development (Kasparian et al., 2016), the trial protocol (Dieng et al., 2015, 2016a), pilot test results (Dieng et al., 2017), main results (Dieng et al., 2016b, 2020), and economic evaluation (Dieng et al., 2019) have been published elsewhere.

Existing frameworks were used to plan, organize, and operationalize process evaluation components, including the UK Medical Research Council framework (Baranowski and Stables, 2000; Moore et al., 2015). Quantitative and qualitative process data were collected using survey instruments, psychologist session notes and audio-recordings, fidelity checklists, the study protocol, and research notes (see Supplementary Materials for a summary of the operationalization of each process evaluation concept and measurement techniques). Data were collected from participants with a history of Stage 0–II melanoma who were current patients of one of three High Risk Melanoma Clinics (HRCs) in Sydney, Australia. Most of the quantitative and qualitative data for the process evaluation (e.g., barriers, satisfaction, dose, contamination) were derived from participant surveys completed at 6-month follow-up. Fear of new or recurrent melanoma was assessed at baseline (pre-randomization), and at 1-, 6- and 12-month follow-up using the 9-item Severity subscale of the validated 42-item Fear of Cancer Recurrence Inventory (FCRI; Simard and Savard, 2009).

### Intervention Description

**Figure 2:**
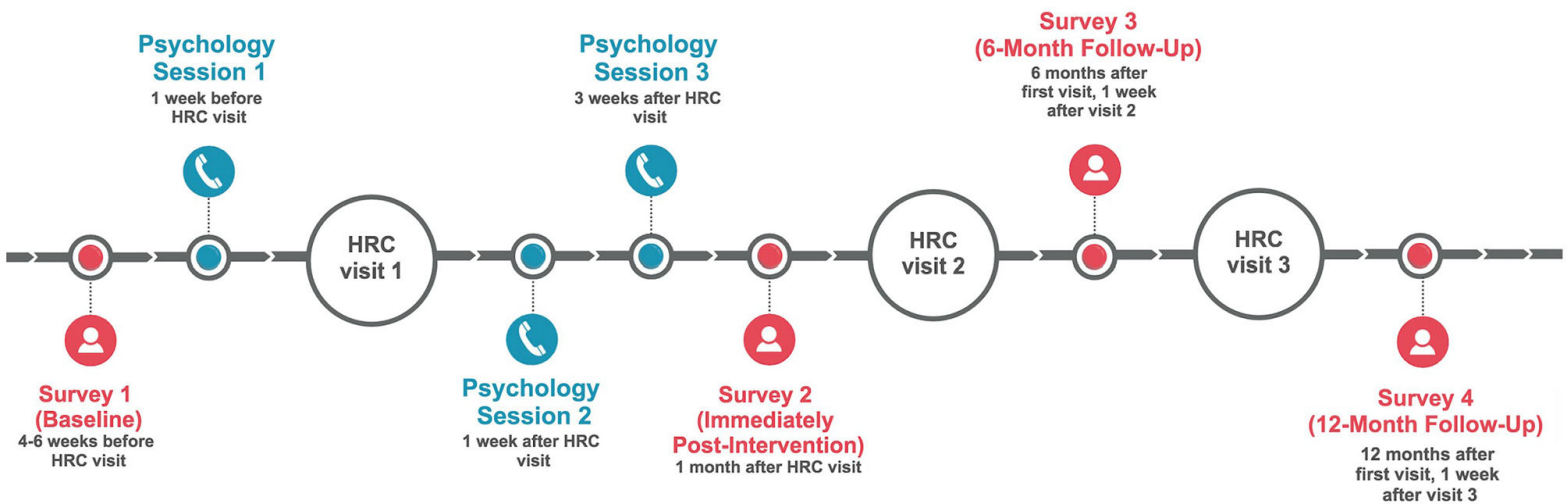
Schedule and timing of individual psychology telephone sessions and study survey assessments. HRC, High Risk Clinic.

The intervention comprised two main components: a newly-developed, 68-page psycho-educational resource provided in color booklet format (called, “*Melanoma: Questions and Answers*”) and three individual psychotherapeutic sessions with a psychologist, delivered *via* telehealth (by telephone) and scheduled in conjunction with patients' dermatology appointment (see Figure 2 for session timing). *Melanoma: Questions and Answers* was developed in response to patient education needs and preferences (McLoone et al., 2012), and includes seven standalone modules covering medical, psychological, behavioral, social, and practical aspects of melanoma, with an emphasis on fear of cancer recurrence. It also includes tools tailored to the needs of people with melanoma, such as graphics to communicate information about melanoma risk, photographs to illustrate complex health behaviors (e.g., skin self-examination), a question prompt list to facilitate doctor-patient communication, verbatim quotes from Australian melanoma patients, care planning tools to record various aspects of melanoma care (such as diagnoses, treatments, moles being monitored for change, and clinical test results), and lists of relevant, reputable services and websites.

In accordance with established evidence (Kasparian, 2013; McLoone et al., 2013a; Kasparian et al., 2016) and brief psychodynamically-oriented psychotherapy principles (Abbass et al., 2009; Shedler, 2010), the overall goals of the psychological intervention sessions were to provide empathic, active listening and to assist participants in fostering strategies to manage health-related distress. Goals of Session 1 (up to 90 min) included discussion and assessment of each participant's background (e.g., family, work, friendships), experience of melanoma and clinical care, information and support needs, and their hopes and goals for the intervention. Sessions 2 and 3 (up to 50 mins each) involved discussing the previous session and each participant's recent dermatology appointment, as well as exploring and addressing individual needs and concerns, and utilizing psychological techniques and components of *Melanoma: Questions and Answers*, as needed. With permission, all sessions were audio-recorded and a detailed summary was prepared by the psychologist immediately after each session.

All participants (intervention and control) also received a copy of the Australian Cancer Council booklet, *Understanding Melanoma*, which includes easy-to-read information about melanoma diagnosis, treatment, and general tips for living well after treatment.

Participants in the control group received usual care, comprising their usual dermatological appointments within the same HRCs and a copy of the Cancer Council booklet, *Understanding Melanoma*.

#### Context of Intervention Delivery

Recruitment occurred at all three high-risk melanoma clinics (HRCs) across the state of New South Wales (Sydney Melanoma Diagnostic Center at the Royal Prince Alfred Hospital, Melanoma Institute Australia in North Sydney, Newcastle Skin Check Clinic); two in metropolitan Sydney and one in regional Newcastle. The clinics provide specialized dermatological care using numerous medical imaging diagnostic interventions for people at high risk of melanoma and patients attend clinics at least 6-monthly (Maloney et al., 2014). To attend, patients need to meet one or more of the following criteria:

1. Previous diagnosis of ≥1 invasive melanoma and dysplastic nevus syndrome;
2. Previous diagnosis of ≥2 invasive melanomas, with at least one diagnosed within the past 10 years;
3. Previous diagnosis of ≥1 invasive melanoma and a family history of ≥3 first-degree or second-degree family members with melanoma; or
4. Carrier of a *CDKN2A* or *CDK4* gene mutation.

Intervention sessions were distance-delivered *via* telephone to overcome geographical barriers to accessing care and to meet previously-identified patient preferences for less travel (McLoone et al., 2013a,b). The first psychotherapeutic session occurred in the week prior to patients' dermatological appointment, when anxiety amongst melanoma patients is known to be highest (Baughan et al., 1993; Morton et al., 2013).

#### Process Evaluation Components: Delivery, Training, and Resources

The psycho-educational resource was developed in partnership with patients and an interdisciplinary team of health professionals and researchers (Kasparian et al., 2016). After comprehensive training that included education on melanoma and clinical management, skills-based training in telehealth for people with cancer, observation of HRC appointments, training in use of the treatment manual and intervention resources, and simulation sessions with a professional actor, three licensed psychologists, each with ≥5 years of clinical experience, delivered the intervention, receiving weekly, distance-delivered (telephone-based) clinical supervision with an experienced psychologist throughout the trial.

#### Intervention Reach, Dose, and Fidelity

As outlined in Baranowski and Stables (2000), reach was defined as the extent to which the intervention contacted or was received by the targeted group. Intervention reach was assessed using enrolment, response, and completion rates, including the proportion of eligible patients who enrolled in the trial, the proportion of enrolled patients who were contacted and received the intervention, and the number of participants who received all intervention components. Sociodemographic characteristics of those who received the intervention were also examined. Dose was defined as the amount of intervention received by participants (i.e., number of sessions and number of minutes spent with the psychologist), and how thoroughly participants reported reading the resources provided on a scale from 0 (“I did not read the booklet”) to 4 (“Read from cover to cover”). Fidelity to the intervention manual (to determine whether the intervention was delivered as intended), was assessed independently by two assessors using a purposively-designed, 24-item checklist (available from authors on request). Initially, the two assessors and a senior supervising psychologist listened to and rated one intervention session individually. Ratings were discussed as a group until consensus was reached and a scoring protocol was devised. For 10% of participants, selected at random and stratified by psychologist, all three intervention session recordings were assessed for fidelity. A sample of these participants (20%) was also used to determine inter-rater reliability, calculated as the percentage of item ratings agreed upon by both assessors.

#### Barriers

Participants were asked to rate the perceived level of difficulty experienced in engaging with intervention components, from 0 (“not at all difficult”) to 10 (“extremely difficult”) in the 6-month follow-up questionnaire. Barriers to intervention implementation were measured from the patient perspective only.

### Mechanisms of Impact

#### Participant Satisfaction

At 6-month follow-up, participants rated their satisfaction with each intervention component on a scale from 0 (“not at all satisfied”) to 10 (“extremely satisfied”). Participants rated overall ***perceived quality of information and support***provided during the study on a scale from 1 (“poor”) to 5 (“excellent”). ***Perceived benefit*** of each component was assessed from 0 (“not at all beneficial”) to 10 (“extremely beneficial”). Participants also rated the degree to which the intervention was felt to have changed various aspects of their life (e.g., improved communication with their clinician, greater understanding of melanoma risk), from 0 (“strongly disagree”) to 4 (“strongly agree”), as well as how helpful they found the different modules and tools provided in *Melanoma: Questions and Answers*. Participants in the control arm responded to the same questions for satisfaction, perceived benefit, and quality of information and support for the Cancer Council *Understanding Melanoma* booklet only.

#### Contamination

To determine whether study outcomes may have been contaminated by access to information outside of the study protocol, participants were asked at 6-month follow-up if they had accessed additional information, and if so, to indicate the sources accessed.

#### Potential Moderators of Intervention Effect

Factors that may have influenced changes in the primary outcome (i.e., change in FCRI Severity scores from baseline to 6-month follow-up) were examined. These factors were: participant sex, time since most recent melanoma diagnosis, baseline FCRI Severity scores, amount of intervention received (i.e., how thoroughly participants reported reading the psychoeducational resource and total duration of psychotherapy sessions), satisfaction with the psychoeducational resource and with psychotherapy sessions, and change in melanoma-related knowledge, as determined by the difference in correctly answered questions (from baseline to 6-month follow-up) on a 9-item melanoma knowledge scale.

### Statistical Analyses

Descriptive statistics were used to examine quantitative aspects of recruitment, reach, fidelity, dose, participant satisfaction, barriers, and contamination. Independent *t*-tests were used to examine potential differences in dose (i.e., how thoroughly participants read the resources provided), satisfaction, and barriers between intervention and control group participants. For the intervention group, paired *t*-tests were used to determine if there were differences in session duration between the psychologists; how thoroughly the resources, *Understanding Melanoma* and *Melanoma: Questions and Answers*, were read; and reported satisfaction and barriers encountered between the two booklet resources and the psychotherapy sessions. Pearson Chi-Squared tests were used to determine potential differences between the intervention and control groups in terms of whether participants would recommend the program to others, as well as whether external information or support was accessed. Multiple linear regression was used for moderator analyses, with sex, time since last melanoma diagnosis, baseline FCRI Severity score, satisfaction with *Melanoma: Questions and Answers*, thoroughness of engagement with the psycho-educational resource, change in melanoma-related knowledge, total duration of psychology sessions, and satisfaction with sessions examined as potential predictors of the primary outcome (i.e., change in FCRI Severity score). Confidence intervals were set to 95%. Quantitative data were analyzed using IBM SPSS Statistics 25 (Armonk, NY: IBM Corp). Qualitative data from participant surveys on satisfaction, perceived benefits, and barriers were analyzed thematically using *NVivo 11*.

## Results

### Intervention Implementation

#### Reach

Of the 346 patients identified as eligible, 183 individuals (53%) consented into the trial. In the period between consent and randomization, 19 participants (10%) withdrew or did not return their baseline questionnaire, leaving 164 participants randomized to the intervention (*n* = 80) or usual care (*n* = 84). Reasons for withdrawal included not needing support (*n* = 5), or lack of time to participate (*n* = 5). Most participants completed the 1-month (87%, *n* = 143) and 6-month (92%, *n* = 151) questionnaires. More men (55%) than women (45%) were recruited into the study, and most participants were from metropolitan areas. The mean participant age was 58.5 ± 11.9 years (Range: 31–83 years). Mean time between last melanoma diagnosis and randomization was 7.6 ± 6.7 years [Range: −0.5 (new diagnosis during study) to 42.6]. Two-thirds (68%) of participants reported FCRI Severity scores of 13 or above at baseline, suggestive of clinically concerning FCR warranting psychological intervention (Simard et al., 2013).

Examining intervention sessions, the first psychologist was assigned to facilitate sessions with 39 participants (49%), second psychologist with 12 participants (15%), and third psychologist with 29 participants (36%). Four of 80 intervention participants (5%) did not take part in the sessions due to not being contactable (*n* = 2), becoming ineligible (*n* = 1), or because they stated that they did not require support (*n* = 1). Three participants in the control arm reported not receiving the Cancer Council *Understanding Melanoma* booklet. All intervention participants reported receiving both *Melanoma: Questions and Answers* and *Understanding Melanoma* booklets.

#### Dose Delivered

Of those who participated in the psychotherapy sessions (*n* = 76), most (*n* = 70) engaged in all three sessions. Four participated in one session and two participated in five sessions due to requiring additional support. Reasons for engagement in only one session included participants feeling they did not require support (*n* = 2), finding talking upsetting (*n* = 1), and becoming unavailable (*n* = 1). Mean session duration (*n* = 76) was 100.7 ± 61.2 min (Range: 2–150 min). When examined by session, the average session length was 53.2 ± 24 min for Session 1, 28 ± 20.7 min for Session 2, and 22 ± 17.8 min for Session 3. Overall, Psychologist 2 facilitated significantly longer sessions (158.9 ± 51.7 min) than Psychologist 1 (96.8 ± 65.5 min, *t*_47_ = −2.99, *p* = 0.004) and Psychologist 3 (79.2 ± 40 min, *t*_36_ = 5.2, *p* < 0.001). No difference in session duration was found between Psychologists 1 and 3 (*t*_60_ = 1.31, *p* = 0.19).

#### Dose Received

Psychologists recorded the content covered in each session (Table 1). Most Session 1 discussions (95%) included an assessment and exploration of the participant's melanoma history. Nearly half the intervention group discussed concerns about their upcoming HRC appointment (48%), and about one-third (30%) discussed types of melanoma and skin self-examination. Much of Session 2 and Session 3 (80–100%) involved reviewing previous sessions as well as participants' experiences of their recent HRC appointment. For one-quarter of participants, Session 2 included discussion of concerns about their next HRC appointment. Resource-related content and unmet information needs were covered more in Session 1 than in Sessions 2 and 3. Tools, such as the skin self-examination guide, mole tracking sheets, and education on coping were more frequently covered in Sessions 2 and 3 than Session 1.

**Table 1:** Content of telehealth-based psychotherapeutic sessions and the proportion of participants who received this content.

**Content**		**Session 1** ***n* = 76**	**Session 2** ***n* = 72**	**Session 3** ***n* = 72**
Assessment and melanoma history and experiences		95%	–	–
Discussion of HRC appointment				
	Concerns around next appointment	48%	25%	14%
	Update of most recent appointment	–	99%	100%
Review of last phone session		–	81%	88%
Resource content (MQA booklet)	Types of melanoma	30%	3%	3%
	Diagram of melanoma diagnosis statistics	13%	1%	3%
	Melanoma and genetic factors	12%	3%	–
	Skin self-examination	30%	4%	4%
	The role of vitamin D	9%	1%	–
	Sun exposure	13%	1%	–
Unmet information needs	Prognosis	21%	3%	–
	Type of melanoma	11%	4%	–
	Genetic risk	12%	–	4%
	Sun exposure/sun screen	17%	1%	–
	Skin self-examination	18%	3%	3%
	Risk to children due to genetic or other factors	24%	1%	1%
Psycho-therapeutic techniques	Worry postponement	5%	8%	6%
	Detached mindfulness	3%	8%	11%
Referral for further psychological care		5%	7%	15%
Sleep		9%	10%	7%
Anxiety		11%	3%	6%
Depression		4%	3%	6%
Stress		8%	10%	4%
Experience of intervention participation and feedback		–	–	85%

*Dash (–) indicates content not covered.*

*MQA, Melanoma: Questions and Answers*.

Overall, intervention participants reported reading *Understanding Melanoma* more thoroughly (*M* = 2.7 ± 1.1 out of 4) than control participants (*M* = 2.27 ± 1.3, *t*_143_ = −2.15, *p* = 0.03). Reasons cited by control participants for not thoroughly reading *Understanding Melanoma* included having enough information about melanoma (*n* = 3) and not feeling worried (*n* = 1). Reasons for intervention participants not reading *Melanoma: Questions and Answers* thoroughly included already having enough information (*n* = 2) and not enjoying reading in general (*n* = 1). Mean ratings for how thoroughly intervention participants read *Understanding Melanoma* (*M* = 2.70 ± 1.13 out of 4) and *Melanoma: Questions and Answers* (*M* = 2.75 ± 1.11 out of 4) did not differ (*t*_66_ = −0.83, *p* = 0.41). Three participants in the intervention group explicitly reported not remembering how thoroughly they read the resources.

#### Fidelity

Overall fidelity to the intervention manual across all three psychotherapeutic sessions was high (88%), with high inter-rater reliability between the two assessors (87%). Fidelity was 83% for Session 1, 88% for Session 2, and 90% for Session 3. Fidelity was 87% for Psychologist, 96% for Psychologist 2, and 86% for Psychologist 3.

#### Barriers

Both groups reported little difficulty engaging with the educational resources. Mean perceived difficulty engaging with *Understanding Melanoma* was very low and did not differ between intervention (*M* = 1.00 ± 1.88 out of 10) and control groups (*M* = 1.45 ± 2.25 out of 10, *t*_141_ = 1.28, *p* = 0.20; Table 2). In the intervention group, mean difficulty engaging with *Understanding Melanoma* (*M* = 1.0 ± 1.88 out of 10) and *Melanoma: Questions and Answers* (*M* = 0.99 ± 1.85 out of 10, *t*_66_ = 0.57, *p* = 0.57) was also low and did not differ. While intervention participants reported little difficulty engaging with the psychotherapy sessions (*M* = 1.8 ± 2.55 out of 10), the mean difficulty rating for the sessions was higher than that for the *Understanding Melanoma* booklet (*t*_64_ = 3.43, *p* = 0.001) and *Melanoma: Questions and Answers* (*t*_64_ = 3.55, *p* = 0.001); however, all difficulty ratings were low. Three participants (4%) found discussing their melanoma experiences with the psychologist confronting, and two participants (3%) reported difficulty finding a suitable time and location to take part in the telehealth sessions.

**Table 2:** Mean perceived satisfaction, benefit, and difficulty of study components. Each component was rated from 0 (“not at all”) to 10 (“extremely”).

**Intervention Component**		**Satisfaction** ***Mean (SD)***	**Perceived Benefit *Mean (SD)***	**Perceived Difficulty** ***Mean (SD)***
*Understanding Melanoma* information booklet	Control group	6.75 (2.61)	6.13 (2.73)	1.45 (2.25)
	Intervention group	7.87 (2.34)	7.52 (2.44)	1.00 (1.88)
*Melanoma: Questions and Answers* psycho-educational resource		7.93 (2.29)	7.48 (2.42)	0.99 (1.85)
Psychotherapeutic sessions *via* telehealth		7.70 (2.81)	7.08 (3.06)	1.80 (2.55)
Overall study participation		7.79 (2.33)	7.28 (2.65)	1.51 (2.44)

“*Melanoma: Questions and Answers psycho-educational resource,” “Psychotherapeutic sessions via telehealth,” and “Overall study participation” refer to those in the intervention group only. Intervention group n = 67, Control group n = 77*.

### Mechanisms of Impact

#### Participant Satisfaction

Satisfaction scores for the Cancer Council *Understanding Melanoma* booklet were significantly higher in the intervention (*M* = 7.87 ± 2.34 out of 10) than control group (*M* = 6.75 ± 2.61 out of 10, *p* = 0.008; Table 2). In the intervention group, no differences were found between mean satisfaction ratings for *Understanding Melanoma* and *Melanoma: Questions and Answers* (*t*_66_ = 0.78, *p* = 0.44). Moreover, mean satisfaction ratings for the telehealth-based psychotherapy sessions (*M* = 7.70 ± 2.81 out of 10) and *Melanoma: Questions and Answers* resource (*M* = 7.93 ± 2.29) were high did not differ significantly (*t*_65_ = −0.58, *p* = 0.57). Perceived quality of information provided in the study was significantly higher in the intervention (*M* = 4.41 ± 0.74 out of 5) than control group (*M* = 3.95 ± 1.07 out of 5; *t*_138_ = −2.94, *p* = 0.004), as was the perceived quality of support (intervention group, *M* = 4.30 ± 0.84 out of 5, control group, *M* = 3.61 ± 1.19 out of 5; *t*_131.3_ = −4.02, *p* < 0.001).

#### Perceived Benefits

Perceived benefit ratings for the *Understanding Melanoma* booklet were significantly higher in the intervention (*M* = 7.52 ± 2.44 out of 10) than control group (*M* = 6.13 ± 2.73 out of 10, *t*_141_ = −3.18, *p* = 0.002; Table 2). Participants who received the intervention rated the benefits of both educational resources, *Understanding Melanoma* (*M* = 7.52 ± 2.44 out of 10) and *Melanoma: Questions and Answers* (*M* = 7.48 ± 2.42 out of 10), highly (*t*_65_ = 0.55, *p* = 0.58). In addition, intervention participants' ratings of the benefits of *Melanoma: Questions and Answers* and the psychology sessions (*M* = 7.08 ± 3.06) did not differ (*t*_65_ = 1.03, *p* = 0.31). Qualitatively, participants spontaneously reported many benefits of the psychological intervention, including improved or reinforced melanoma-related knowledge (*n* = 5), increased risk awareness (*n* = 4) and health behaviors (*n* = 2), and better communication with their clinician and family members (*n* = 2). Ten participants referred to the intervention as “informative”, while others (*n* = 3) believed the knowledge gained empowered better coping and decision-making, and one participant reported increased optimism regarding survival. Eight participants spontaneously reported deriving benefit from the psychotherapy sessions and three participants mentioned sharing the resources with others. Some participants (*n* = 4) believed the intervention would have been more beneficial if provided at the time of melanoma diagnosis, and others (*n* = 7) believed it would be more beneficial for individuals with higher levels of melanoma-related worry.

In survey responses, participants identified improved communication with their melanoma care team as the greatest benefit of the intervention (Table 3). Other perceived benefits included learning how often to check their skin, greater understanding of their risk of recurrence, and feeling less worried about HRC appointments.

**Table 3:** Perceived effects of participation in the intervention (*n* = 67), with ratings from 0 (“strongly disagree”) to 4 (“strongly agree”).

**Participating in this psychological study has helped me to:**	***Mean (SD)***
Talk more openly with my High Risk Melanoma Clinic doctor and ask questions when I need to	2.90 (0.89)
Know how often it is recommended that I check my skin	2.88 (0.75)
Better understand my risk of developing another melanoma	2.87 (0.78)
Know more about the recommended ways to check my skin and what to look	2.85 (0.74)
Feel less worried about my High Risk Melanoma Clinic appointments	2.63 (0.81)
Find the information I need to cope as best as I can with melanoma	2.63 (0.76)
Talk more openly with my family about melanoma	2.60 (0.78)
Feel more confident in my ability to cope with worries or concerns I have about melanoma	2.58 (0.74)
Understand why I feel the way I do about my melanoma risk	2.57 (0.74)
Feel more confident in my ability to cope with worries or concerns in general	2.46 (0.75)
Find the emotional support I need to cope as best as I can with melanoma	2.40 (0.84)
Find other services that may be helpful for me or my family	2.39 (0.72)
Feel more confident to use coping strategies such as Detached Mindfulness	2.31 (0.82)
Get emotional help and support about issues unrelated to my melanoma risk	2.21 (0.75)

Information presented in *Melanoma: Questions and Answers* rated most helpful included information about the different types of melanoma (*M* = 2.35 ± 0.81 out of 3) and about how to monitor moles for changes (*M* = 2.35 ± 0.76 out of 3; Table 4). The most used and most helpful tool in *Melanoma: Questions and Answers* was the Skin Self-Examination Guide, used by 73% of intervention participants and perceived as helpful by 71% of those who used it (Table 5). The least used tool was the SunSmart App (19% usage); this and the Appointment Calendars were perceived as the least helpful tools.

**Table 4:** Mean perceived helpfulness ratings for each component of *Melanoma: Questions and Answers* (*n* = 66), with ratings from 0 (“not at all helpful”) to 3 (“very helpful”).

**Psychoeducational resource component**	**Perceived helpfulness *Mean (SD)***
Different types of melanoma	2.35 (0.81)
Monitoring moles for change	2.35 (0.76)
Risk of developing melanoma presented in 100-person risk diagrams	2.21 (0.83)
Skin self-examination	2.33 (0.74)
Genetics and family history	2.17 (0.80)
Sun protection after a melanoma diagnosis	2.18 (0.88)
Vitamin D	2.09 (0.86)
How melanoma can affect the way I feel	1.91 (0.87)
Coping with melanoma	1.92 (0.94)
Living with the fear that melanoma may come back	1.74 (0.87)
Quotes and messages from people who have had melanoma	1.62 (0.96)

**Table 5:** Reported use and perceived helpfulness of the tools included in *Melanoma: Questions and Answers* (*n* = 69).

**Tool**	**Percentage of participants who reported use**	**Percentage of participants who perceived tool as helpful**
Skin self-examination guide	73%	71%
Moles and spots record	62%	62%
UV index explainer	44%	54%
Question prompt list	46%	44%
Menu of coping strategies	38%	44%
Checklist for recognizing signs of stress	32%	38%
Future melanoma appointments	29%	38%
List of useful websites and services	26%	38%
Diagnosis and treatment record	26%	35%
Appointment calendars	25%	30%
SunSmart app	19%	30%

Over 80% of intervention participants reported they would recommend the intervention to other people with melanoma, and 82% of control group participants would recommend *Understanding Melanoma*. The two groups did not differ in terms of whether they would recommend the program to others [$\chi_{\left(2,141\right)}^{2}$ = 1.12, *p* = 0.57].

### Moderators

Multiple linear regression was used to examine a range of potential moderators of intervention effect; however, only one of the hypothesized moderators (baseline FCRI Severity score) was found to predict the primary outcome (i.e., change in FCRI Severity scores from baseline to 6-month follow-up). Higher FCRI Severity scores at baseline were associated with a greater decrease in FCR severity at 6-month follow-up (Table 6). Overall, the model accounted for only 7% of the variance in the primary outcome.

**Table 6:** Multiple linear regression examining potential moderators of intervention effect (*n* = 61).

**Factor**	**Unstandardized coefficient**	**95% CI**	**Standardized coefficient**	***p*-value**	**Unique variance accounted for (%)**
Participant sex	−0.65	−3.24, 1.95	−0.07	0.620	0.41
Time since last melanoma diagnosis	0.00	−0.02, 0.01	−0.03	0.853	0.06
Baseline FCRI Severity score	−0.17	−0.33, −0.01	−0.28	**0.044**	7.02
Satisfaction with the psycho-educational resource, M*elanoma: Questions and Answers*	0.00	−0.58, 0.58	0.00	0.991	0.00
Thoroughness of engagement with the psycho-educational resource	0.05	−1.13, 1.23	0.01	0.930	0.01
Change in melanoma-related knowledge	0.03	−0.21, 0.26	0.03	0.816	0.09
Total duration of psychotherapy sessions	0.00	−0.02, 0.02	0.00	0.984	0.00
Satisfaction with psychotherapy sessions	0.17	−0.29, 0.63	0.11	0.462	0.90

*Significant results in bold typeface. CI, confidence interval*.

### Contamination

External sources of information accessed by participants during the study included internet sites, their general practitioner, and family and friends. Access to additional information sources did not differ between groups, with 19 (28%) intervention participants and 18 control participants (24%) reporting use of additional information sources [$\chi_{\left(1,143\right)}^{2}$ = 0.41, *p* = 0.52]. Key findings from the process evaluation are summarized in Table 7.

**Table 7:** Summary of the main findings of the process evaluation.

**Intervention implementation**	**Main findings**
Reach	• Of the 346 eligible patients, 183 (53%) consented into the trial. In the time between consent and randomization, 19 participants (10%) withdrew or did not return their baseline questionnaire. • 164 participants randomized to the intervention (*n* = 80) or usual care (*n* = 84). • Most participants completed the 1-month (87%, *n* = 143) and 6-month (92%, *n* = 151) assessments. • More men (55%) than women (45%) were recruited, and most participants were from metropolitan areas. Mean participant age was 58.5 ± 11.9 years (Range: 31–83 years). • Mean time between last melanoma diagnosis and randomization was 7.6 ± 6.7 years. • Two-thirds (68%) of participants reported FCRI Severity scores of 13 or above at baseline, suggestive of clinically concerning FCR warranting psychological intervention. • Four of 80 intervention participants (5%) did not participate in the psychotherapy sessions.
Dose delivered	• Of those who participated in the sessions (*n* = 76), most (*n* = 70) engaged in all three sessions. • Mean session length was 53.2 ± 24 min for Session 1, 28 ± 20.7 min for Session 2, and 22 ± 17.8 min for Session 3. • Overall, Psychologist 2 facilitated significantly longer sessions than Psychologist 1 and Psychologist 3.
Dose received	• Most Session 1 discussions (95%) included assessment and exploration of the participant's melanoma history, as well as concerns about one's upcoming HRC appointment. • Much of Session 2 and Session 3 (80–100%) involved reviewing previous sessions and participants' experiences of their recent HRC appointment • Intervention participants reported reading *Understanding Melanoma* more thoroughly than control participants. • Mean ratings for how thoroughly intervention participants read *Understanding Melanoma* and *Melanoma: Questions and Answers* did not differ.
Fidelity	• Fidelity of the psychotherapeutic sessions to the intervention manual was high (88%), with high inter-rater reliability between the two assessors (87%). • Fidelity was 83% for Session 1, 88% for Session 2, and 90% for Session 3.
Barriers	• Both groups reported little difficulty engaging with the educational resources. • Engaging in the psychotherapy sessions was perceived as more difficult than engaging with the informational and psycho-educational resources; however, all difficulty ratings were low.
**Mechanisms of impact**	**Main findings**
Participant satisfaction	• Satisfaction scores for the Cancer Council *Understanding Melanoma* booklet were significantly higher in the intervention than control group. • Perceived quality of information provided throughout the study was significantly higher in the intervention than control group, as was perceived quality of support.
Perceived benefits	• Perceived benefit ratings for the *Understanding Melanoma* booklet were significantly higher in the intervention than control group. • Reported benefits of the psychological intervention included improved or reinforced melanoma-related knowledge, increased risk awareness and health behaviors, and better communication with participants' clinician and family members. • Participants identified improved communication with their melanoma care team as the greatest benefit.
Moderators	• Only one of the hypothesized factors—baseline FCRI Severity score—was found to moderate intervention effect (i.e., change in FCRI Severity scores from baseline to 6-month follow-up), with higher FCRI Severity scores at baseline associated with a greater decrease in FCR severity at 6-month follow-up.
Contamination	• External sources of information accessed by participants during the study included internet sites, participants' general practitioner, and family and friends. Reported access to additional information sources did not differ between groups.

## Discussion

To our knowledge, this is the first process evaluation of a psychological intervention targeting fear of cancer recurrence, and one of very few in cancer. Results complement and strengthen the published outcomes of the original clinical trial by demonstrating that the intervention was delivered as intended and was acceptable, feasible, and very well received by participants. Attrition throughout the trial was considerably lower than other published trials of FCR interventions involving in-person sessions (e.g., ConquerFear; Butow et al., 2017), which could be due in part to our intervention being more accessible and suggests that telehealth-based interventions are a feasible and potentially preferred alternative to interventions delivered in-person. Nearly all participants used the psycho-educational resource, engaged in all psychotherapy sessions, and reported high satisfaction with both of these intervention components. Difficulty ratings were very low, indicating limited barriers to engaging with the intervention. Over 80% of participants would recommend the intervention to others and identified numerous benefits that may have direct positive impact on their experience of melanoma and clinical care, with the most highly rated benefits being enhanced doctor-patient communication, talking more openly with family members about melanoma, and improved coping. Trial outcomes appear to be due to direct effects of the intervention and were unlikely due to external factors. While external sources of information were accessed by some participants, this did not differ between intervention and control groups, indicating low contamination and providing increased confidence that improved FCR outcomes were a result of intervention participation.

The results of this process evaluation augment the outcomes of the RCT and provide indications as to why the intervention was effective. One possible “active ingredient” is increased engagement with melanoma-related information. Intervention participants reported reading the Cancer Council booklet, *Understanding Melanoma*, more thoroughly than participants in usual care, which may partially explain why intervention participants reported higher satisfaction and greater benefit from the booklet than control participants. While change in melanoma-related knowledge was not a moderator of intervention effect, the brief measure we used to assess knowledge may not have captured other informational benefits gained from the resources. Another “active ingredient” could be increased confidence in one's ability to manage melanoma. This is supported by findings in the intervention group that information about melanoma, moles, and skin self-examination in *Melanoma: Questions and Answers* was rated most helpful and used most often. Additionally, the most commonly reported benefits of the intervention included being able to talk more openly with one's doctor, knowing more about how and when to perform skin checks, and better understanding one's of risk of melanoma recurrence. Finally, receiving tailored support from a trained and experienced psychologist could be an “active ingredient.” Intervention participants reported receiving significantly better quality of information and support compared with the control group, indicating that the intervention provided benefit over and above resources available as part of usual care. The finding that intervention participants reported reading the Cancer Council booklet, *Understanding Melanoma*, more thoroughly than the control group may reflect an effect of the psychologists referring to resource content during sessions, prompting and supporting participants to utilize and meaningfully engage with the resources more. Future studies comparing provision of the psycho-educational resource (*Melanoma: Questions and Answers*) alone vs. coupled with psychotherapy sessions could provide further clarity on the role of the therapist in psychological interventions for people with melanoma.

The process evaluation also offers rich information about how participants engaged with the intervention. Satisfaction with and reported benefits of the two resources were similar amongst intervention participants, indicating the newly-developed resource, *Melanoma: Questions and Answers*, was as acceptable as the pre-existing resource, *Understanding Melanoma*, despite being 16-pages longer. While the briefer information provided by the Cancer Council booklet was well accepted by both groups, our findings suggest it alone was insufficient in addressing patients' needs. Overall, these results support a combination of psychologist-assisted and self-directed activities (e.g., reading informational and psycho-educational resources) was more favorably perceived than the informational booklet alone and led to greater psychological benefits for patients.

Intervention participants were provided with a large amount of medical and psychological information, as well as psycho-educational tools, and covered a range of topics within psychotherapy sessions. Information about melanoma, moles, and skin self-examination were rated the most helpful and most used components of the resource, aligning with research showing that health-related information is one of the greatest unmet needs reported by people with melanoma and other cancers (Beesley et al., 2015; Sarkar et al., 2015; Stamataki et al., 2015; Fu et al., 2020; Mutsaers et al., 2020). The SunSmart App was the least used and least helpful tool, which may reflect the preferences of the older demographic of our study (Lim, 2010). Calendars were also not perceived as very useful and participants may already employ other strategies to keep track of appointments. Although the psychology sessions followed a clear framework, session scope and content were tailored to participants' specific goals, preferences, needs, and difficulties. Areas most frequently covered in sessions, and therefore more likely to be important to participants, included reflection on one's experiences with melanoma, information about the different types of melanoma, discussion of skin self-examination, and working through worries and concerns about upcoming dermatology appointments. Less commonly discussed topics included anxiety, depression, and specific psychotherapeutic techniques, such as mindfulness. This may reflect a relatively lower need for specific psychological strategies, especially as some participants may have entered the study with relatively low FCR, and a greater need for accurate information about melanoma and an empathic health professional with which to discuss one's experiences.

We examined a range of potential moderators of intervention effects (e.g., participant sex, amount of intervention received, time since last melanoma diagnosis, baseline FCRI Severity scores, and satisfaction with the intervention) and found that baseline (pre-intervention) FCR severity was the only significant moderator. Higher FCR severity at baseline was associated with a greater decrease in FCR severity at 6-months post-intervention. This suggests that people with higher FCR were more likely to derive benefit from the intervention, and that the intervention was successful in targeting the primary outcome of interest (i.e., FCR; Dieng et al., 2016b). The data did not support our hypothesis that participants who received a greater dose of the intervention (i.e., longer duration with the psychologist, more thorough engagement with *Melanoma: Questions and Answers*) would report a greater decrease in FCR than those who were less engaged with the intervention. In addition, while information was perceived by many as a helpful component of the intervention, change in melanoma-related knowledge was not related to change in FCR severity. Previous research has shown that psycho-educational interventions can lower distress amongst people with melanoma (McLoone et al., 2013a) and that supportive psychotherapy, where participants have opportunities to discuss the issues most important to them, can reduce distress in other cancer populations (Classen et al., 2001; Breitbart et al., 2018). Overall, our findings suggest that regardless of factors such as participant sex, intervention dose, and time since last melanoma diagnosis, the intervention had the greatest impact on those who reported the greatest need.

### Study Limitations

Several limitations warrant discussion. Three participants (4%) reported not remembering how thoroughly they read their resources, as the survey was administered 6 months after receiving the booklets, suggesting recall bias may have affected the accuracy of process evaluation results (Bowling, 2005). The moderator analysis showed that baseline FCRI Severity scores, a participant characteristic and not a process component, was the only factor that predicted change in FCRI Severity scores. Although our results suggest potential benefits of pre-intervention screening, baseline FCRI Severity scores were analyzed as a continuous variable in the regression and it was beyond the scope of this study to determine what constitutes a “high” baseline score. Data on the proportion of psychotherapy sessions that covered various topics or themes provided insight into the nature and scope of the sessions; however, further information could be gathered on potential patterns in session content and themes, the total time spent discussing different themes, and whether this affected intervention outcomes. While this analysis would be time-consuming and resource heavy, it could provide highly valuable insights into specific mechanisms of, or pathways to, psychotherapeutic outcomes and effects.

Barriers that may have impeded intervention implementation were measured only from the patient perspective, limiting our understanding of other factors that may have influenced treatment engagement. Future research is needed to explore barriers experienced by psychologists when facilitating and delivering psychological interventions, as well as systemic and environmental factors, to better understand the feasibility of implementing this and other psychological interventions in routine clinical practice. Finally, most of the researchers involved in the design, implementation, and outcome evaluation of the intervention were also involved in the process evaluation and were not blinded to treatment condition. This may have introduced potential biases in how data were presented and interpreted. Separating process evaluation and outcome evaluation teams could be considered in future research. Pros and cons of separating or integrating process and outcome evaluation teams have been discussed elsewhere (Moore et al., 2015).

### Recommendations for Translation into Clinical Practice and Future Research

Process evaluations are an important adjunct to outcome studies as they enhance researchers' ability to interpret intervention outcomes and provide valuable evidence to inform translation into clinical practice. The results of this process evaluation support a number of recommendations (Table 8) for effective delivery of a psychological intervention targeting fear of cancer recurrence amongst melanoma patients.

**Table 8:** Clinical recommendations based on outcomes of the process evaluation.

**Outcome**	**Clinical recommendations**
The psychological intervention was delivered as intended (high fidelity) and was well-received by participants.	• Implementation of the psychological intervention, into routine clinical care for Stage 0–II melanoma patients at high risk of new primary disease. • Assess fear of cancer recurrence and unmet information and support needs, and offer the intervention based on patient need and preference.
Satisfaction and perceived benefits of the educational resources (*Melanoma: Questions and Answers* and *Understanding Melanoma*) were high.	• In resource-limited environments, offering two resources to patients—one that provides brief information about melanoma (i.e., *Understanding Melanoma*) and one that contains detailed and tailored psychoeducation (i.e., *Melanoma: Questions and Answers*)—is a low-cost initial step to addressing unmet information and support needs.
Participants who received the intervention reported greater satisfaction with the Cancer Council booklet, compared with participants who received usual care. Intervention participants also perceived the quality of information and support throughout the study as greater than participants in the control group.	• Psychologist support is recommended in conjunction with informational and psycho-educational resources. • Psychologists can assist patients in utilizing and engaging with resource content, which may lead to higher levels of satisfaction with care. • A patient-centered psychological intervention including written information and opportunities to discuss and reflect on experiences with a trained mental health professional offers best practice care to patients.
Number and duration of telehealth sessions with a psychologist varied between participants in the intervention group.	• Up to three psychology sessions is likely to be sufficient for most Stage 0-III melanoma patients. • At least 3 sessions can be offered, though the number and duration of sessions should be tailored to each individual patient and should be timed around upcoming dermatological appointments (see Figure 2).
Nearly all intervention participants engaged in the psychology sessions. Distance-delivered sessions provided *via* telehealth were acceptable and rated low in terms of difficulty.	• Trained mental health professionals such as psychologists should be included as part of the clinical care team. • Tailored, patient-centered psychotherapy sessions should be implemented as part of routine care, and should provide opportunities to discuss patients' experiences of melanoma as well as their worries about upcoming clinic visits. • Distance-delivered interventions, provided *via* telehealth, minimize common barriers to accessing mental health care and are highly acceptable to melanoma patients.
Baseline fear of cancer recurrence was the only significant moderator of intervention effect.	• Routine FCR screening for all Stage 0–II melanoma patients prior to dermatological appointments may assist in identifying those people likely to derive the greatest benefit from interventions targeting FCR. • Wide dissemination of the intervention is strongly recommended, as factors such as participant sex, age, and time since melanoma diagnosis did not influence efficacy.

Our findings demonstrate the ways in which future trials would benefit from inclusion of a structured process evaluation. In addition, we offer the following recommendations for future research:

- This study showed that people with higher levels of FCR at baseline reported the greatest decrease in FCR post-intervention; however, there is currently no consensus regarding what cut-off score constitutes “high” FCR, nor what score is indicative of referral for intervention. Brief measurement tools have shown promise and may reduce the cognitive burden on patients while quickly identifying those with greatest clinical need (Fardell et al., 2018; Rudy et al., 2020; Smith et al., 2020). Further research to identify a clinically-meaningful FCR cut-off score for people affected by melanoma will allow for pre-intervention screening and more precise targeting of treatments and interventions (Lebel et al., 2017). In terms of our intervention, having a clearly defined clinical cut-off score would also facilitate future research examining whether offering the intervention to those with clinical levels of FCR only would lead to different results and “active ingredients.”
- Psycho-educational resources should include information and tools relating to different types of melanoma, moles, and skin self-examination, while appointment calendars and Smartphone apps may be optional or omitted, depending on patient age, smartphone use, and type of app.
- Intervention delivery by experienced psychologists who received tailored training and ongoing supervision proved highly successful in this study. Other implementation models, such as in-person or videoconference sessions with a psychologist, cancer nurse, or social worker, could also be considered in future research.
- The effectiveness of a stepped-care model, where resources are allocated based on patient preference and level of need, could also be explored. All patients could be offered screening using an FCR measure and then provided with the psycho-educational resource as a first step, followed by telehealth-based psychotherapy sessions for those with greater need. At a minimum, our findings suggest telehealth-based sessions should include discussions about the patient's melanoma experience, unmet information needs, and concerns regarding upcoming dermatology appointments.

### Conclusions

Overall, the findings of this process evaluation confirm that the published psychological intervention aimed at reducing FCR amongst individuals at high-risk of developing another melanoma was feasible and highly effective, implemented as intended, very well-received by participants, and led to numerous benefits for participants. Results suggest the “active ingredients” of our intervention included increased engagement with melanoma-related information, and highly accessible support from a trained and experienced mental health professional. The clearest recommendation for implementation into routine care is FCR screening to identify those most likely to derive the greatest benefit from intervention referral; however, given the lack of consensus regarding a clinical cut-off score in this population and our observation that participants may derive other health and educational benefits, we cannot conclude that this intervention should be provided only to patients who report “high” FCR without further investigation. Guidelines and suggestions for how FCR can be managed in clinical practice have been outlined elsewhere (Butow et al., 2018; Mutsaers et al., 2020). General recommendations follow a stepped care approach, including routine use of an FCR measure (especially at the end of cancer treatment and during follow-up appointments), and provision of psychoeducational resources and sessions with a trained mental health professional, when indicated. Implementation in clinical settings is a vital next step if we are to provide all melanoma patients with the opportunity to access care that is person-centered and meets their medical, informational and psychological needs.

## Data Availability Statement

The raw data supporting the conclusions of this article will be made available by the authors, without undue reservation.

## Ethics Statement

The studies involving human participants were reviewed and approved by Sydney Local Health District Ethics Review Committee, Department of Health and Ageing Human Research Ethics Committee, the University of Sydney Human Research Ethics Committee, and the Australian Institute of Health and Welfare Ethics Committee. The patients/participants provided their written informed consent to participate in this study.

## Author Contributions

NK, AC, MD, PB, DC, RM, SWM, SM, and GM: conception and design. AC, NK, and GM: financial support. NK and SWM: provision of study materials or patients. MD, AC, NK, and ST: collection and assembly of data. JK, DC, NK, SM, and ST: data analysis and interpretation. JK, NK, ST, MD, DC, PB, SWM, RM, GM, and AC: manuscript writing. All authors contributed to the article and approved the submitted version.

## Conflict of Interest

The authors declare that the research was conducted in the absence of any commercial or financial relationships that could be construed as a potential conflict of interest.

## References

[B1] Abbass A. Kisely S. Kroenke K. (2009). Short-term psychodynamic psychotherapy for somatic disorders. Psychother. Psychosomatics 78, 265–274. 10.1159/000228247 19602915

[B2] Baranowski T. Stables G. (2000). Process evaluations of the 5-a-day projects. Health Educ. Behav. 27, 157–166. 10.1177/109019810002700202 10768797

[B3] Baughan C. Hall V. Leppard B. Perkins P. (1993). Follow-up in stage I cutaneous malignant melanoma: an audit. Clin. Oncol. 5, 174–180. 10.1016/S0936-6555(05)80321-8 8347541

[B4] Beesley V. L. Smithers B. M. Khosrotehrani K. Khatun M. O'Rourke P. Hughes M. C. B.. (2015). Supportive care needs, anxiety, depression and quality of life amongst newly diagnosed patients with localised invasive cutaneous melanoma in Queensland, Australia. Psycho Oncol. 24, 763–770. 10.1002/pon.3718 25355178

[B5] Bowling A. (2005). Mode of questionnaire administration can have serious effects on data quality. J. Public Health 27, 281–291. 10.1093/pubmed/fdi031 15870099

[B6] Breitbart W. Pessin H. Rosenfeld B. Applebaum A. J. Lichtenthal W. G. Li Y.. (2018). Individual meaning-centered psychotherapy for the treatment of psychological and existential distress: a randomized controlled trial in patients with advanced cancer. Cancer 124, 3231–3239. 10.1002/cncr.31539 29757459 PMC6097940

[B7] Butow P. N. Sharpe L. Thewes B. Turner J. Gilchrist J. Beith J. (2018). Fear of cancer recurrence: a practical guide for clinicians. Oncology 32, 32–38. 29447419

[B8] Butow P. N. Turner J. Gilchrist J. Sharpe L. Smith A. B. Fardell J. E.. (2017). Randomized trial of ConquerFear: a novel, theoretically based psychosocial intervention for fear of cancer recurrence. J. Clin. Oncol. 35, 4066–4077. 10.1200/JCO.2017.73.1257 29095681

[B9] Classen C. Butler L. D. Koopman C. Miller E. DiMiceli S. Giese-Davis J.. (2001). Supportive-expressive group therapy and distress in patients with metastatic breast cancer: a randomized clinical intervention trial. Arch. General Psychiatry 58, 494–501. 10.1001/archpsyc.58.5.494 11343530

[B10] Costa D. S. J. Dieng M. Cust A. E. Butow P. N. Kasparian N. A. (2016). Psychometric properties of the fear of cancer recurrence inventory: an item response theory approach. Psycho Oncol. 25, 832–838. 10.1002/pon.4018 26489770

[B11] Crist J. V. Grunfeld E. A. (2013). Factors reported to influence fear of recurrence in cancer patients: a systematic review. Psycho Oncol. 22, 978–986. 10.1002/pon.3114 22674873

[B12] Dieng M. Butow P. N. Costa D. S. J. Morton R. L. Menzies S. W. Mireskandari S.. (2016b). Psychoeducational intervention to reduce fear of cancer recurrence in people at high risk of developing another primary melanoma: results of a randomized controlled trial. J. Clin. Oncol. 34, 4405–4414. 10.1200/JCO.2016.68.2278 27998215

[B13] Dieng M. Cust A. E. Kasparian N. A. Butow P. N. Costa D. S. J. Menzies S. W.. (2016a). Protocol for a within-trial economic evaluation of a psycho-educational intervention tailored to people at high risk of developing a second or subsequent melanoma. BMJ Open 6:e012153. 10.1136/bmjopen-2016-012153 PMC5073649 27855094

[B14] Dieng M. Kasparian N. Mireskandari S. Butow P. Costa D. Morton R.. (2017). Psychoeducational intervention for people at high risk of developing another melanoma: a pilot randomised controlled trial. BMJ Open 7:e015195. 10.1136/bmjopen-2016-015195 29018064 PMC5652456

[B15] Dieng M. Kasparian N. A. Morton R. L. Mann G. J. Butow P. N. Menzies S.. (2015). The Melanoma care study: protocol of a randomised controlled trial of a psycho-educational intervention for melanoma survivors at high risk of developing new primary disease. BMC psychol. 3, 1–13. 10.1186/s40359-015-0074-3 26167282 PMC4499167

[B16] Dieng M. Khanna N. Kasparian N. A. Costa D. S. Butow P. N. Menzies S. W.. (2019). Cost-effectiveness of a psycho-educational intervention targeting fear of cancer recurrence in people treated for early-stage melanoma. Appl. Health Econ. Health Policy 17, 669–681. 10.1007/s40258-019-00483-6 31228015

[B17] Dieng M. Morton R. L. Costa D. S. J. Butow P. N. Menzies S. Lo S.. (2020). Benefits of a brief psychological intervention targeting fear of cancer recurrence in people at high risk of developing another melanoma: 12-month follow-up results of a randomized controlled trial. Br. J. Dermatol. 182, 860–868. 10.1111/bjd.17990 30965384

[B18] Fardell J. E. Jones G. Smith A. B. Lebel S. Thewes B. Costa D.. (2018). Exploring the screening capacity of the fear of cancer recurrence inventory-short form for clinical levels of fear of cancer recurrence. Psycho Oncol. 27, 492–499 10.1002/pon.4516 28755462

[B19] Fu H. Teleni L. Crichton M. Chan R. J. (2020). Supportive care and unmet needs in patients with melanoma: a mixed-methods systematic review. Suppor. Care Cancer 28, 3489–3501. 10.1007/s00520-020-05464-3 32342223

[B20] Kasparian N. A. (2013). Psychological care for people with melanoma: what, when, why and how? Semin. Oncol. Nurs. 29, 214–222. 10.1016/j.soncn.2013.06.007 23958219

[B21] Kasparian N. A. Mireskandari S. Butow P. N. Dieng M. Cust A. E. Meiser B.. (2016). “Melanoma: questions and answers.” Development and evaluation of a psycho-educational resource for people with a history of melanoma. Support Care Cancer 24, 4849–4859. 10.1007/s00520-016-3339-3 27465047

[B22] Koch L. Jansen L. Brenner H. Arndt V. (2013). Fear of recurrence and disease progression in long-term (≥5 years) cancer survivors—a systematic review of quantitative studies. Psycho Oncol. 22, 1–11. 10.1002/pon.3022 22232030

[B23] Lebel S. Mutsaers B. Tomei C. Leclair C. S. Jones G. Petricone-Westwood D.. (2020). Health anxiety and illness-related fears across diverse chronic illnesses: a systematic review on conceptualization, measurement, prevalence, course, and correlates. PLoS ONE 15:e0234124. 10.1371/journal.pone.0234124 32716932 PMC7384626

[B24] Lebel S. Ozakinci G. Humphris G. Thewes B. Prins J. Dinkel A.. (2017). Current state and future prospects of research on fear of cancer recurrence. Psycho Oncol. 26, 424–427. 10.1002/pon.4103 26891602

[B25] Lebel S. Tomei C. Feldstain A. Beattie S. McCallum M. (2013). Does fear of cancer recurrence predict cancer survivors' health care use? Support. Care Cancer 21, 901–906. 10.1007/s00520-012-1685-3 23269420

[B26] Lim C. S. C. (2010). Designing inclusive ICT products for older users: taking into account the technology generation effect. J. Eng. Design 21, 189–206. 10.1080/09544820903317001

[B27] Maloney F. J. Guitera P. Coates E. Haass N. K. Ho K. Khoury R.. (2014). Detection of primary melanoma in individuals at extreme high risk a prospective 5-year follow-up study. JAMA Dermatol. 150, 819–827. 10.1001/jamadermatol.2014.514 24964862

[B28] McLoone J. K. Menzies S. Meiser B. Mann G. J. Kasparian N. A. (2013a). Psycho-educational interventions for melanoma survivors: a systematic review. Psycho Oncol. 22, 1444–1456. 10.1002/pon.3165 22933380

[B29] McLoone J. K. Watts K. Menzies S. Barlow-Stewart K. Mann G. Kasparian N. (2013b). Melanoma survivors at high risk of developing new primary disease: a qualitative examination of the factors that contribute to patient satisfaction with clinical care. Psycho Oncol. 22, 1994–2000. 10.1002/pon.3243 23382138

[B30] McLoone J. K. Watts K. Menzies S. Meiser B. Butow P. N. Kasparian N. A. (2012). When the risks are high: psychological adjustment among melanoma survivors at high risk of developing new primary disease. Qualitative Health Res. 22, 1102–1113. 10.1177/1049732312448542 22673092

[B31] Moore G. Audrey S. Barker M. Bond L. Bonell C. Cooper C.. (2014). Process evaluation in complex public health intervention studies: the need for guidance. J. Epidemiol. Community Health 68, 101–102. 10.1136/jech-2013-202869 24022816 PMC3892708

[B32] Moore G. Audrey S. Barker M. Bond L. Bonell C. Hardeman W.. (2015). Process evaluation of complex interventions: Medical Research Council guidance. Br. Med. J. 350:h1258. 10.1136/bmj.h1258 25791983 PMC4366184

[B33] Morton R. L. Rychetnik L. McCaffery K. Thompson J. F. Irwig L. (2013). Patients' perspectives of long-term follow-up for localised cutaneous melanoma. Euro. J. Surg. Oncol. 39, 297–303. 10.1016/j.ejso.2012.12.017 23287820

[B34] Mutsaers B. Jones G. Rutkowski N. Tomei C. Leclair C. S. Petricone-Westwood D.. (2016). When fear of cancer recurrence becomes a clinical issue: a qualitative analysis of features associated with clinical fear of cancer recurrence. Support. Care Cancer 24, 4207–4218. 10.1007/s00520-016-3248-5 27169700

[B35] Mutsaers B. Rutkowski N. Jones G. Lamarche J. Lebel S. (2020). Assessing and managing patient fear of cancer recurrence. Can. Family Phys. 66, 672–673. 32933983 PMC7491663

[B36] Rudy L. Maheu C. Körner A. Lebel S. Gélinas C. (2020). The FCR-1: initial validation of a single-item measure of fear of cancer recurrence. Psycho Oncol. 29, 788–795. 10.1002/pon.5350 32026563

[B37] Sarkar S. Sautier L. Schilling G. Bokemeyer C. Koch U. Mehnert A. (2015). Anxiety and fear of cancer recurrence and its association with supportive care needs and health-care service utilization in cancer patients. J. Cancer Survivorship 9, 567–575. 10.1007/s11764-015-0434-2 25676473

[B38] Shedler J. (2010). The efficacy of psychodynamic psychotherapy. Am. Psychol. 65, 98–109. 10.1037/a0018378 20141265

[B39] Simard S. Savard J. (2009). Fear of cancer recurrence inventory: development and initial validation of a multidimensional measure of fear of cancer recurrence. Support Care Cancer 17, 241–251. 10.1007/s00520-008-0444-y 18414902

[B40] Simard S. Thewes B. Humphris G. Dixon M. Hayden C. Mireskandari S.. (2013). Fear of cancer recurrence in adult cancer survivors: a systematic review of quantitative studies. J. Cancer Survivorship 7, 300–322. 10.1007/s11764-013-0272-z 23475398

[B41] Smith A. B. Costa D. Galica J. Lebel S. Tauber N. van Helmondt S. J.. (2020). Spotlight on the Fear of Cancer Recurrence Inventory (FCRI). Psychol. Res. Behav. Manage. 13:1257. 10.2147/PRBM.S231577 33376421 PMC7762428

[B42] Stamataki Z. Brunton L. Lorigan P. Green A. C. Newton-Bishop J. Molassiotis A. (2015). Assessing the impact of diagnosis and the related supportive care needs in patients with cutaneous melanoma. Support. Care Cancer 23, 779–789. 10.1007/s00520-014-2414-x 25189151

