# Supplementary material for: Identifying the ‘Active Ingredients' of an Effective Psychological Intervention to Reduce Fear of Cancer Recurrence: A Process Evaluation
Source: Front Psychol. 2021 Jun 7;12:661190. doi: 10.3389/fpsyg.2021.661190 (PMC8215538; doi:10.3389/fpsyg.2021.661190)
Supplement: Supplementary file 1 [file Table_1.DOCX]

**SUPPLEMENTARY MATERIALS**

**Elements of the process evaluation, operationalisation of each concept, and measurement techniques.**

| **COMPONENT** | **OPERATIONALISATION** | **MEASURES OR DATA SOURCES** |
| --- | --- | --- |
| **CONTEXT** | | |
| **CONTEXT**  Aspects of the environment of the intervention. | - Contextual factors: - HRC environment - Timing of psychology sessions in relation HRC appointments - Ability of participants to take part in the intervention from home | - Research protocol, psychotherapy manual, and HRC observations |
| **IMPLEMENTATION** | | |
| **DELIVERY, TRAINING AND RESOURCES** | - Resource factors, such as providing training to psychologists who delivered the intervention about melanoma, HRCs, and the intervention protocol | - Research protocol and intervention protocol |
| **RECRUITMENT**  Attracting participants to the intervention. | - Resources utilised to attain participation. - Number of potential participants. - Number of actual participants. - Number of people who refused or were not contactable, and reasons for refusal. - Differences between recruited sample and study population on selected characteristics. | - Research protocol - HRC clinical databases - Research database |
| **REACH**  Extent to which the target group was contacted and received the intervention. Proportion of the target sample who participated in the intervention. | - Spread: number (or proportion) of participants who received each component of the intervention (quantitative), and sociodemographic patterning. - Depth: aspects or components of the intervention received (qualitative). | - Enrolment, response, and completion rates. - Psychologist files and research database - Psychologist session audio-recordings |
| **DOSE**  ***Completeness (dose delivered)***  **+ *Exposure (dose received)***  Extent of active engagement and receptiveness of the intervention by participants. Exposure is a characteristic of the participants and assesses the extent of their engagement. | - Number of intended units of each intervention component delivered or provided by interventionists. - Proportion of the psycho-educational resource read or otherwise used by participants. - Number and duration of each psychology session participant took part in. - Content of each psychology session participant took part in. - Types of activities carried out by interventionists and participants. - Number of activities carried out by interventionists and participants. | - Psychologist files, research database, and session audio-recordings |
| **FIDELITY**  Extent to which the intervention was implemented as designed and planned. Fidelity represents the quality and integrity of the intervention as conceived by the developers, and is a function of the intervention providers. | - Extent to which psychologists followed the intervention delivery protocol and used materials designed for use in the intervention (i.e. manual, risk assessment and referral process). | - Psychologist files, research database, and session audio-recordings |
| **BARRIERS**  Problems encountered while applying the intervention and reaching participants. | - Barriers in applying the intervention. - Reasons or causes for deviation from protocol. | - Psychologist and researcher notes, and feedback provided in participant surveys |
| **MECHANISMS OF IMPACT** | | |
| **PARTICIPANT RESPONSES TO AND INTERACTIONS WITH THE INTERVENTION**  Participants’ satisfaction with the intervention | - Participants’ overall experience of the intervention. - Perceived benefits (use, helpfulness) of each intervention component. - Perceived burdens (limitations, risks) associated with each intervention component. - Overall perceived usefulness of the intervention. | - Participant survey for all aspects of satisfaction, including both quantitative and qualitative responses |
| **MODERATORS** |  |  |
| **CONTAMINATION**  Extent to which participants received interventions outside of the program and extent to which the control group received treatment. | - Types of components of competing programs reaching participants. - Types of components of programs reaching control group participants. - Number of competing programs reaching participants. | - Participant surveys |
